# Factors associated with pertussis infection and suspected cases in the Aseer Region, Saudi Arabia: a retrospective cross-sectional analysis study

**DOI:** 10.3389/fpubh.2026.1685920

**Published:** 2026-02-12

**Authors:** Abdulrahman Mohammed Alqahtani, Abdullah Mohammed Alshehri, Arif Ahmed Alqahtani, Majd Mohammed Yahya Asiri, Maram Mohammed Alshahrani

**Affiliations:** 1 Department of Preventive Medicine, Aseer Health Cluster, Aseer Region, Abha City, Saudi Arabia; 2 Department of Preventive Medicine, Ministry of Health, Aseer Region, Abha City, Saudi Arabia; 3 Department of Preventive Medicine, Armed Forces Hospital, Khamis Mushait, Saudi Arabia; 4 Department of Public Health, Ministry of Health, Aseer Region, Abha City, Saudi Arabia

**Keywords:** Aseer region, Bordetella pertussis, epidemiology, pediatric, pertussis, Saudi Arabia, vaccination, whooping cough

## Abstract

**Background:**

Pertussis is a major cause of vaccine-preventable morbidity and mortality in children globally. In Saudi Arabia, the incidence has risen despite high immunization rates and maternal Tdap recommendations. This study aims to identify the incidence and key factors associated with laboratory-confirmed pediatric pertussis in the Aseer region from 2020 to 2024.

**Methods:**

A retrospective analysis was conducted on suspected pediatric pertussis cases at Sarat Ubaida General Hospital, Aseer region, from 2020 to 2024. Data from medical records included age, gender, vaccination history, symptoms, and lab results. Descriptive statistics summarized characteristics, with Chi-square and Fisher's Exact tests for comparisons, and multivariable logistic regression for adjusted odds ratios (95% CI).

**Results:**

Of 388 suspected cases, 41.5% tested positive. Infants under four months made up 58.3% of cases, and males accounted for 51.3%. Fewer than 42% had received any pertussis vaccine, with only 28.6% of mothers receiving Tdap in the third trimester. Annual positivity dropped from 37.1% in 2020 to 5.9% in 2022, then rose to 69.3% in 2024. Maternal Tdap immunization significantly reduced positivity rates: 38.9% in unvaccinated mothers compared to 77.8% in vaccinated mothers (*p* = 0.003). Classic respiratory symptoms helped distinguish positive from negative cases.

**Conclusion:**

Pediatric pertussis in the Aseer region has seen low vaccination coverage and a resurgence in 2024, especially in infants under four months. It's crucial to boost maternal Tdap uptake, improve infant vaccination access, target low-coverage communities, and maintain surveillance to monitor trends and vaccine effectiveness.

## 1 Introduction

Pertussis is a respiratory disease caused by the bacterium Bordetella pertussis, commonly referred to as whooping cough. A key feature of classic pertussis is the extended duration of coughing, which can last for weeks. This illness is marked by severe coughing fits, known as paroxysms, that typically end with a characteristic gasping noise referred to as a whoop (1) Pertussis impacts millions of people around the world each year, significantly affecting children the most (2). According to the World Health Organization, this disease leads to more than 160,000 fatalities each year, especially among infants under six months old who lack complete vaccination (3).

Pertussis can be prevented through vaccination. The introduction of the pertussis vaccine during the 20th century has led to a notable decrease in the incidence and mortality of this disease among children (4) Despite this advancement, pertussis remains a significant public health issue, with substantial numbers of cases and fatalities still recorded (5) The CDC reports that about one-third of infants diagnosed with pertussis need hospitalization, and around 1% of these hospitalized infants die from the illness (6) Infants are particularly vulnerable to pertussis, being the most affected age group and accounting for nearly all related hospitalizations and deaths. While whooping cough can impact people of any age, it is especially dangerous for young children (7).

The Saudi Ministry of Health focuses on maternal and child health by providing resources for safe pregnancy and childbirth (8) They recommend hexavalent vaccines for infants at two, four, and six months, with boosters at 18 months and preschool entry (9) The Mother and Child Health Passport System tracks health history and routine check-ups (8) Pregnant women should receive the Tdap vaccine during the third trimester, between 27 and 36 weeks (10) From 2016 to 2019, pertussis cases in Saudi Arabia rose from 0.022 to 0.75 per 100,000. In 2019, the western region had 26.5% of the total 68 cases. Vaccination data since 1980 shows high coverage (94%-99% for the first dose) except for the low rates in 1980 (53%) and 1985 (81%) (11) In 2019, infant vaccination coverage was 97% (12).

The serious complications of whooping cough, such as pneumonia, encephalopathy, and potential death, pose urgent risks to children's health, particularly in resource-limited areas with restricted access to vaccines and medical care (13) Despite extensive vaccination efforts, the disease remains a significant global health threat, leading to severe respiratory issues in infants and young children (14, 15) Periodic outbreaks highlight the challenges in controlling its spread. The Aseer region lacks sufficient data on vaccination histories and clinical presentations, which complicates the development of effective public health strategies. This study aims to analyze the incidence and key factors of laboratory-confirmed pediatric pertussis in Aseer from 2020 to 2024, to inform targeted prevention interventions.

## 2 Materials and methods

### 2.1 Study subjects and data collection

The study was conducted through a retrospective analysis of medical records related to suspected pertussis cases at Sarat Ubaida General Hospital in the Aseer region of Saudi Arabia, covering five years from 2020 to 2024. Suspected pertussis cases were defined according to the national surveillance case definition, which includes individuals presenting with a cough lasting ≥2 weeks, accompanied by at least one of the following: paroxysms of coughing, inspiratory whoop, or post-tussive vomiting, without other apparent cause. Sarat Ubaida General Hospital was selected as the study site because it functions as a regional referral center, receiving cases from multiple surrounding primary healthcare facilities. Additionally, the hospital was chosen due to its comprehensive medical records and the accessibility of patient files for the specified study period. All children suspected of having pertussis at Sarat Ubaida General Hospital in the Aseer region, with available medical records from the study period, were included in the analysis. Records that lacked key variables in test results were excluded.

Laboratory confirmation of pertussis was performed using polymerase chain reaction (PCR) testing at the regional Public Health Laboratory. In some cases, infants are presented with other respiratory symptoms but not the classic pertussis cough; these cases were initially tested using multiplex PCR, and when the result was positive for Bordetella pertussis, a confirmatory pertussis-specific PCR sample was collected and sent to the Public Health Laboratory.

Two independent reviewers extracted data using a pre-structured tool to ensure consistency. Data collection included demographic information and vaccination history for each suspected pertussis case, along with a comprehensive set of clinical features. For all records, the reviewers noted whether pertussis was laboratory-confirmed and whether the patient exhibited key symptoms, such as cough, shortness of breath, vomiting, dyspnea, and pneumonia. They also recorded any documented convulsive episodes or neurological complications. Maternal vaccination status was defined as documented receipt of Tdap vaccine during pregnancy, as recorded in antenatal care records.

All data were anonymized before entry and analysis. All children suspected of having pertussis with available medical records at Sarat Ubaida General Hospital in the Aseer region, Saudi Arabia, from 2020 to 2024.

### 2.2 Statistical analysis

The data were entered into an Excel sheet for review and then transferred to SPSS (Statistical Package for the Social Sciences) version 26 for analysis. Descriptive analysis was conducted to summarize categorical data using both numerical and percentage values. An estimated annual incidence rate was calculated by dividing the number of laboratory-confirmed pertussis cases by the corresponding pediatric population of the Aseer region. The relationship between demographic characteristics and clinical features associated with laboratory-confirmed pertussis was assessed using the Chi-square test and Fisher's Exact test. Additionally, multivariable logistic regression was used to estimate adjusted odds ratios (ORs) and 95% confidence intervals (CIs) for factors associated with laboratory-confirmed pertussis. A significant level with a P-value of 0.05 was established. A valid percentage was calculated for missing cases.

### 2.3 Ethical approval

Ethical approval was obtained from the Asir Region Ethics Committee on 13 January 2025 (IRB Log No: E7-1-2025). An official copy of the approval letter, confirming the Ministry of Health as the implementing authority and Sarat Ubaida General Hospital as the study site. All information from study participants was kept confidential and not shared outside the research team. No personal identifiers or names were collected.

## 3 Results

### 3.1 Demographic characteristics and clinical manifestations

The demographic data are shown in Table 1. A total of 388 suspected cases of pertussis, with the majority (58.3%) being infants under 4 months of age. Approximately half of the cases involved males (51.3%). Among the children with available vaccination data, only 41.8% had received at least one pertussis-containing vaccine, and of those children, the majority (62.6%) had received only one dose. Additionally, maternal Tdap vaccination during the third trimester was reported for only 28.6% of the 63 mothers with available data. In our study, under-vaccination was a significant concern. Only 41.8% of infants received their first DTaP dose, and just 28.6% of mothers received a Tdap booster during the third trimester. As a result, infants were left vulnerable, lacking direct immunity and missing out on passive antibody transfer. This situation increased their susceptibility and likely contributed to the high rate of laboratory-confirmed pertussis observed in the obtained findings.

**Table 1:** Demographic profile and vaccination status of suspected pediatric pertussis cases (*n* = 388).

**Factors**	**Number**	**Percentage**
**Age**		
< 2 months	72	18.6
2–3 months	154	39.7
4–6 months	65	16.8
7–12 months	49	12.6
> 12 months	48	12.4
**Gender**		
Female	189	48.7
Male	199	51.3
**Vaccination (*****N*** = **371)**		
Yes	155	41.8
No	216	58.2
**Vaccination times (*****N*** = **155)**		
One dose	97	62.6
Two doses	18	11.6
Three doses	40	25.8
One dose	97	62.6
**Maternal vaccination details (*****N*** = **63)**		
Yes, Tdap, 3rd trimester, MOH clinic	18	28.6
No, not vaccinated	45	71.4

Of 388 Suspected Pediatric Cases, 41.5% (*n* = 161) tested positive for pertussis, while 58.5% (*n* = 227) were negative.

Among 388 suspected pediatric cases, the recorded symptoms included cough (47.7%), shortness of breath (27.1%), vomiting (25.0%), dyspnea (24.5%), and radiographically confirmed pneumonia (34.5%). Notably, 41.5% (*n* = 161) of these children had confirmed pertussis, as shown in Table 2.

**Table 2:** Clinical features of suspected pediatric pertussis cases (*n* = 388).

**Factors**	**Number**	**Percentage**
**Confirmed pertussis cases**		
Positive	161	41.5
Negative	227	58.5
**Cough**		
Yes	185	47.7
No	203	52.3
**Shortness of breath**		
Yes	105	27.1
No	283	72.9
**Vomiting**		
Yes	97	25.0
No	291	75.0
**Dyspnea**		
Yes	95	24.5
No	293	75.5
**Pneumonia**		
Positive	134	34.5
Negative	254	65.5
**Convulsions**		
Yes	6	1.5
No	382	98.5
**Brain problems**		
Yes	1	0.3
No	387	99.7

### 3.2 Epidemiological data on suspected and confirmed pertussis cases (2020–2024)

Between 2020 and 2024, testing for pertussis in children varied. In 2020, 70 children were tested, resulting in a positivity rate of 37.1%. This rate dropped to 21.6% in 2021 and further declined to 5.9% in 2022. In 2023, the rate rose to 15.1%, but in 2024, with 163 children tested, the positivity rate increased dramatically to 69.3%, as shown in Table 3.

**Table 3:** Epidemiological data on suspected and confirmed pertussis cases (2020–2024).

**Year**	**Total**	**Positive test result for pertussis**	
		**Number**	**Percentage**
2020	70	26	37.1
2021	51	11	21.6
2022	51	3	5.9
2023	53	8	15.1
2024	163	113	69.3

### 3.3 Correlation between patients' demographic data and confirmed pertussis

No significant association was observed between confirmed pertussis diagnosis and the infant's age, gender, or basic vaccination status. Although the number of vaccine doses received by infants offered protection, this did not achieve statistical significance (*p* = 0.059). In contrast, the timing and completeness of maternal Tdap (tetanus, diphtheria, and pertussis) immunization during the third trimester proved to be very protective: infants whose mothers received the Tdap vaccine at a Ministry of Health clinic during the third trimester had significantly lower rates of pertussis positivity (38.9% vs. 77.8%; *p* = 0.003). Clinical features were effective in distinguishing between positive and negative cases: 55.7% of infants with cough tested positive compared to 28.6% of those without a cough (*p* < 0.001); 61.0% of those with shortness of breath tested positive, vs. 34.3% without (*p* < 0.001); 52.6% with vomiting tested positive compared to 37.8% without (*p* = 0.011); and 60.0% of infants with dyspnea tested positive, compared to 35.5% without (*p* < 0.001), as presented in Table 4.

**Table 4:** Analysis of predictors for confirmed pertussis cases.

**Factors**	**Confirmed pertussis cases number (percentage)**		***p*-value**
	**Positive**	**Negative**	
**Age**			
< 4 months	88 (38.9)	138 (61.1)	0.227
≥4 months	73 (45.1)	89 (54.9)	
**Gender**			
Female	83 (43.9)	106 (56.1)	0.346
Male	78 (39.2)	121 (60.8)	
**Vaccination**			
Yes	61 (39.4)	94 (60.6)	0.248
No	98 (45.4)	118 (54.6)	
**Vaccination times**			
One dose	44 (45.4)	53 (54.6)	0.059
Two doses	3 (16.7)	15 (83.3)	
Three doses	14 (35.0)	26 (65.0)	
**Maternal vaccination details**			
Yes, Tdap, 3rd trimester, MOH clinic	35 (38.9)	11 (61.1)	**0.003**
No, not vaccinated	35 (77.8)	10 (22.2)	
**Cough**			
Yes	103 (55.7)	82 (44.3)	**< 0.001**
No	58 (28.6)	145 (71.4)	
**Shortness of breath**			
Yes	64 (61.0)	41 (39.0)	**< 0.001**
No	97 (34.3)	186 (65.7)	
**Vomiting**			
Yes	51 (52.6)	46 (47.4)	**0.011**
No	110 (37.8)	181 (62.2)	
**Dyspnea**			
Yes	57 (60.0)	38 (40.0)	**< 0.001**
No	104 (35.5)	189 (64.5)	
**Pneumonia**			
Positive	57 (42.5)	77 (57.5)	0.762
Negative	104 (40.9)	150 (59.1)	
**Convulsions**			
Yes	3 (50)	3 (50)	0.696^*^
No	158 (41.4)	224 (58.6)	
**Brain problems**			
Yes	0 (0)	1 (100)	1^*^
No	161 (41.6)	226 (58.4)	

^*^Fisher's Exact Test. Bold values indicate statistically significant results (*p* < 0.05).

### 3.4 Multivariable model for predictors of confirmed pertussis

Ages under four months, being female, any completed vaccine dose, and absence of respiratory signs all indicated lower odds of laboratory-confirmed pertussis. Meanwhile, a lack of maternal Tdap vaccination was associated with higher odds; however, none of these findings reached statistical significance.

## 4 Discussion

Despite the broad implementation of vaccination programs, there has been a noticeable increase in pertussis cases in recent years, revealing significant hurdles in global vaccination efforts. Contributing factors include decreased immunity resulting from the use of acellular vaccines, the emergence of different strains of Bordetella pertussis, and the underreporting of mild cases in older children and adults. These issues help sustain the ongoing spread of the disease, particularly putting unvaccinated or inadequately immunized infants at higher risk (16, 17) Our study aimed to investigate the epidemiology of suspected pediatric pertussis cases in Aseer from 2020 to 2024 and to identify demographic, maternal, vaccination, and clinical predictors of laboratory-confirmed infection.

This analysis provides critical insights into the epidemiology of pertussis in the Aseer region. The data indicates a significant disease burden among infants under 4 months of age, with an incidence rate of 58.3%. Additionally, 41.5% of the 388 suspected cases of pediatric pertussis were confirmed through laboratory tests. This emphasizes the considerable impact of pertussis infections in symptomatic children. Classic respiratory symptoms, including cough, shortness of breath, dyspnea, and vomiting, were strong and significant indicators that helped distinguish between positive and negative cases. This underscores the diagnostic value of these classic clinical signs and symptoms. In Iran, approximately 63.2% of the cases of pediatric leg swelling were found in infants who were less than 6 months old among those hospitalized (18) In Tunisia, infants under 2 months of age represented approximately 48.3% to 56.7% of the confirmed pertussis cases (19, 20) Moreover, two studies highlighted the age demographics of children younger than 6 months. In Oman, 52.9% of the cases involved infants aged 2 months or younger, while 89.2% were 4 months or younger (21) In Iran, approximately 77.5% of cases were found in infants less than 3 months old (22) Additionally, a research study examined 119 cases of mild pertussis and 64 cases of severe pertussis. The severe cases were marked by an earlier onset, a spasmodic cough, difficulty in breathing, and the presence of rales (23).

In our study, Maternal Tdap immunization provided significant protection, with positivity rates of 38.9% among unvaccinated mothers compared to 77.8% among vaccinated mothers. Studies have indicated that the effectiveness of a single dose of the DTP vaccine is only 46%, which is significantly lower than the 91.7% protection offered by a complete series of three doses. This highlights the necessity of ensuring that children receive the entire vaccination series by the age of six months, as recommended by the WHO (24) Research conducted in Saudi Arabia revealed that only 3.7% of women had received the pertussis vaccine during their prior pregnancies, and only 7.0% reported that their healthcare providers had advised them to get the vaccine. Additionally, 44.9% of the participants expressed their willingness to accept the vaccine during pregnancy if it were offered at no cost (25) Concerns regarding vaccine safety have been identified as the main reason for delays in immunization. Additionally, factors such as misinformation, logistical challenges like access to clinics, and historical mistrust in healthcare systems contribute to vaccine hesitancy. To address these issues, enhancing community engagement through culturally relevant educational initiatives that stress vaccine safety, and the concept of herd immunity could help alleviate these challenges (26) To reduce pertussis burden, prioritize maternal Tdap vaccination in third-trimester care, supported by provider training and patient education. Strengthen infant immunization systems to ensure the timely completion of the DTP series through effective outreach and reminder systems. Additionally, combat vaccine hesitancy with culturally responsive campaigns that address safety concerns and emphasize the benefits of collective immunity to rebuild trust in health systems. Several limitations should be noted. First, our focus was on a single region (Aseer), which limits the ability to generalize findings to other areas in Saudi Arabia, or to global contexts that face different healthcare access, vaccination practices, or patterns of pertussis epidemiology. Second, the retrospective design may introduce data bias, as some symptoms or vaccination details might be undocumented, and there may be unmeasured factors that could influence the results. Despite these limitations, the findings offer valuable insights for Aseer. It is crucial to prioritize Tdap vaccination for mothers in the third trimester through integrated antenatal programs. Additionally, improving the timeliness of DTaP vaccinations for infants is important. Implementing real-time surveillance during outbreaks, using identified clinical indicators such as cough and dyspnea, is also recommended. Finally, conducting multi-regional studies would help validate the generalizability of these findings.

## 5 Conclusion

Our five-year surveillance reveals that, despite broad childhood immunization efforts, pertussis is reemerging in the Aseer region, with a 69% positivity rate in 2024. The key modifiable factor for protection is the maternal Tdap vaccine administered in the third trimester; yet, fewer than one-third of mothers receive it. Classic symptoms such as cough and shortness of breath can effectively differentiate pertussis from other causes of infant respiratory distress, indicating that symptom-based screening could improve early detection in primary care. To address this issue, we recommend integrating Tdap into antenatal care, enhancing infant vaccine uptake through outreach, optimizing real-time surveillance, developing clinical decision support tools, and researching vaccination barriers. These interventions are crucial for preventing outbreaks and protecting infants who are too young for full immunization.

## Data Availability

The original contributions presented in the study are included in the article/supplementary material, further inquiries can be directed to the corresponding author.
